# Genome-Wide Association Analysis Revealed Candidate Genes Related to Early Growth Traits in Inner Mongolia Cashmere Goats

**DOI:** 10.3390/vetsci12030192

**Published:** 2025-02-20

**Authors:** Youjun Rong, Xiaofang Ao, Furong Guo, Xinle Wang, Mingxuan Han, Lu Zhang, Qincheng Xia, Fangzheng Shang, Qi Lv, Zhiying Wang, Rui Su, Yanhong Zhao, Yanjun Zhang, Ruijun Wang

**Affiliations:** 1 College of Animal Science, Inner Mongolia Agricultural University, Hohhot 010018, China; rongyoujun1@163.com (Y.R.); aoxiaofang666@163.com (X.A.); 15147807909@163.com (F.G.); wangxinle163@163.com (X.W.); hanmx1997@163.com (M.H.); xxxxxxqc@163.com (Q.X.); sfznnd2020@126.com (F.S.); lvqi1202@foxmail.com (Q.L.); wzhy0321@126.com (Z.W.); suruiyu@126.com (R.S.); 13947196432@163.com (Y.Z.); 2 Science and Technology Development Center of Ulanqab, Ulanqab 012000, China; zl63412732@163.com; 3 Key Laboratory of Mutton Sheep Genetics and Breeding, Ministry of Agriculture, Hohhot 010018, China; 4 Key Laboratory of Goat and Sheep Genetics, Breeding and Reproduction in Inner Mongolia Autonomous Region, Hohhot 010018, China

**Keywords:** Inner Mongolia cashmere goats, genome-wide association analysis, birth weight, weaning weight, haplotype

## Abstract

The Inner Mongolia cashmere goat is a local breed valued for both its cashmere and meat production. Early growth traits include birth weight and weaning weight. Generally, cashmere goats with higher birth and weaning weights tend to exhibit a higher carcass weight at slaughter. Consequently, exploring the genetic variation associated with these early growth traits is crucial in enhancing the meat production performance of Inner Mongolia cashmere goats. For this study, we performed genome-wide association analysis and haplotype analysis on the early growth traits of 212 Inner Mongolia cashmere goats (72 rams, 140 ewes) to identify molecular markers and haplotypes significantly associated with birth and weaning weights. Through genome-wide association analysis and gene annotation, we identified 21 SNPs and 117 candidate genes linked to early growth traits. Notably, genes such as *RUNX1T1*, *ERBIN*, *MYO15B*, *NT5C*, *GRB2*, *ITGB4*, and *GALK* emerged as significant factors influencing the early growth of Inner Mongolia cashmere goats. Additionally, nine haplotype blocks related to early growth traits were constructed, resulting in eight haplotype combinations. In conclusion, the haplotype combinations A1A1, C1C1, and D1D1 were found to be beneficial for the genetic improvement of early growth traits in Inner Mongolia cashmere goats.

## Simple Summary

Inner Mongolia cashmere goats are renowned for their high meat production, distinctive mutton flavor, and tender texture, making them widely favored by consumers. Investigating the genetic variation associated with the early growth traits of Inner Mongolia cashmere goats is crucial in enhancing their meat production. In this study, we identified 21 SNPs and 117 candidate genes related to early growth traits and constructed nine haplotype blocks associated with these traits, resulting in eight haplotype combinations. This research lays a solid foundation for the genetic improvement of early growth traits in Inner Mongolia cashmere goats.

## 1. Introduction

China is rich in a variety of cashmere goat resources, mainly distributed in arid and semi-arid areas and desert and semi-desert areas in Northeast China, Northwest China, and the Qinghai–Tibet Plateau [1], including the Hanshan White cashmere goat, Qaidam cashmere goat, Shanbei White cashmere goat, Hexi cashmere goat, Liaoning cashmere goat, Inner Mongolia cashmere goat, and other excellent varieties. Among these, Inner Mongolia cashmere goats (IMCGs) can be divided into the Erlangshan type, Albas type, and Alashan type according to their origin [2]. The Inner Mongolia cashmere goat (Erlangshan type) is mainly distributed in the Front, Middle, and Rear Banner of Urat, Dengkou County, Bayannur City. Its physique is strong, its neck width is moderate, its back and waist are straight, its chest is wide and deep, and its limbs are solid and strong. These characteristics allow it to still have strong adaptability under harsh natural conditions and ecological environments. In addition, the goat’s mutton has a unique flavor, tender meat, and no odor and has high economic value [3].

Whole-genome sequencing (WGS) involves the deep sequencing of various individuals based on the reference genome sequence of known species, followed by the analysis of genetic differences among individuals to obtain comprehensive genotype information [4]. Genome-wide association studies (GWASs) perform population-level statistical analyses of genotypes and economic traits, identifying genetic variants significantly associated with target traits and subsequently uncovering genes linked to these traits [5,6]. To date, candidate genes related to growth traits have been identified in several goat breeds. The PITX2 gene is significantly correlated with body height and body length traits in Kanto dairy goats and Hainan black goats [7], while the PRDM6 gene influences growth traits such as chest depth, chest width, body length, and body height in cashmere goats [8]. Some researchers have identified 21 SNPs associated with traits such as birth weight, weaning weight, and first-year weight in Inner Mongolia cashmere goats (Albas type), noting that genes closely related to muscle growth, including *MAPK3*, *LDB2*, and *LRP1B*, are enriched in the actin cytoskeleton and phospholipase D signaling pathways [9]. Additionally, genes such as *STPIP2*, *C7orf57*, *CCL19*, *FGF9*, *SGCG*, *FIGN*, and *SIPA1L* have been identified in Dazu black goats as being correlated with traits including body height, body length, tube circumference, chest depth, chest width, and chest circumference [10]. Furthermore, genes such as *CRADD*, *HMGA2*, *MSRB3*, *MAX*, *HACL1*, and *RAB15* have been shown to regulate body size, fat deposition, and average daily weight gain in Karachai goats [11]. Despite these valuable findings, the genetic mechanisms underlying early growth in Inner Mongolia cashmere goats (Erlangshan type) remain insufficiently understood. Therefore, it is crucial to explore the genes that may be involved in the regulation of early growth.

Haplotype blocks are defined as continuous and stable regions of haplotypes on chromosomes that are minimally disrupted by recombination. Consequently, haplotype blocks constructed at significant loci in a linked state may offer a more comprehensive explanation of phenotypic variation and possess greater predictive power than individual SNP markers [12,13,14]. GWAS and haplotype analyses provide an effective approach to screening molecular markers to elucidate the association between genetic variation and complex traits. This understanding is beneficial in enhancing the genetic traits of animals, improving breeding efficiency, and advancing the field of animal genetics and breeding science.

In this study, the Inner Mongolia cashmere goat (Erlangshan type) was selected as the research subject. GWASs on birth weight and weaning weight, along with linkage disequilibrium and haplotype analysis, was conducted to identify new candidate SNPs and genes for the genetic improvement of early growth traits in Inner Mongolia cashmere goats.

## 2. Materials and Methods

### 2.1. Source of Experimental Animal and Phenotype Data

**Figure 1:**
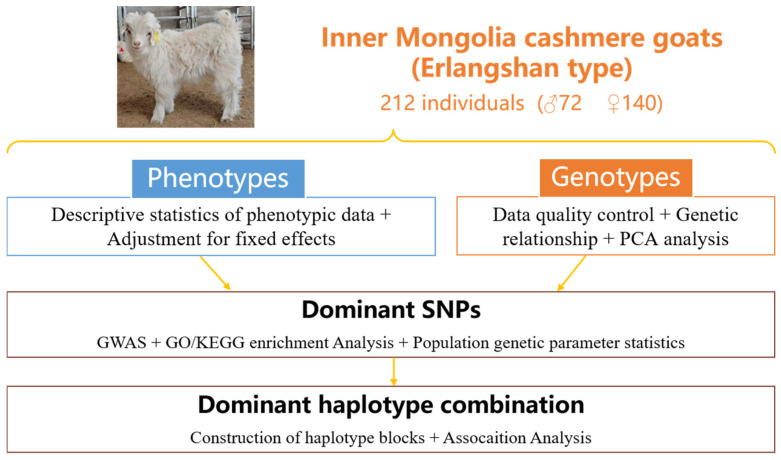
Schematic illustration of the workflow applied to detect SNPs and haplotype combinations that are significantly associated with early growth traits in IMCGs.

In this study, the production performance measurement data were all from Erlangshan Ranch, Inner Mongolia Beiping Textile Co., Ltd., Urat Middle Banner, Bayannur, Inner Mongolia, China. (an Inner Mongolia cashmere goat (Erlangshan type) national protected breeding farm), and the goats were raised under group management, with a total of 9 herds; male and female goats were raised in herds. The live weight of kids before colostrum consumption within 1 h after birth was recorded as the birth weight. Breeding usually starts around October each year, lambing begins around March of the following year, and unified weaning is carried out from June to July. The live weight of a weaned goat on an empty stomach in the morning was recorded as the weaning weight. All experimental animals involved in these studies were managed, fed, and utilized in strict accordance with the relevant animal welfare regulations and ethical guidelines, ensuring their proper care and well-being throughout the experimental procedures. The traits examined included the birth weight and weaning weight of 212 Inner Mongolia cashmere goats (Erlangshan type), comprising 72 bucks and 140 does. Among them, 183 kids were born as singletons, 27 kids were born as twins, and 2 kids were born as triplets. The analysis pipeline is presented in Figure 1. To account for the variability in the number of weaning days among individuals, a formula was developed to standardize the weaning weight to 90 days, as follows [15]:

WW90 = (WW − BW)/WD × 90 + BW

where WW90 is the adjusted weight for 90 days of age. WW is the weaning weight; BW is the birth weight; and WD is the number of weaned days.

### 2.2. Genomic DNA Extraction, Library Construction, and Online Sequencing

Ear tissue samples from 212 Inner Mongolia cashmere goats were collected using ear-cutting forceps (Qingdong Dongjifeng Animal Husbandry Technology Co., Ltd., Qingdao, China.) and subsequently placed in liquid nitrogen for transportation to the laboratory, where they were preserved at −80 °C. The DNA was isolated from these ear tissue specimens by implementing the phenol–chloroform extraction protocol. The integrity and purity of the extracted DNA were evaluated with a spectrophotometer (NanoDrop2000, Thermo Fisher Scientific, Waltham, USA) and agarose gel electrophoresis techniques. The DNA samples that met the quality criteria were stored in a −20 °C freezer (Henan Xinfei Electric Appliance Co., Ltd., Xinxiang, China.) for subsequent experimental applications. Following the processing of the qualified genomic DNA samples, the genomic DNA was randomly fragmented into 350 bp length segments using the Covaris (Covaris, Inc., Woburn, MA, USA) ultrasonic crusher. The complete library was prepared through terminal repair, poly(A) addition, sequencing splice, purification, and PCR amplification. Following the completion of library fabrication, the Qubit 2.0 instrument was applied for an initial assessment of the quantity. Subsequently, quantitative polymerase chain reaction (qPCR) was utilized to accurately measure the effective concentration of the library, aiming to ensure its quality. Once the quality of the library was validated, sequencing was executed on the DNBSEQ-T7 sequencing platform (20×) in the paired-end 150-base-pair (PE150) sequencing format.

### 2.3. Single-Nucleotide Polymorphism Calling

The raw read data were processed and filtered into clean read data by making use of the fastp software (V0.20.0) [16]. The Burrows–Wheeler Aligner (BWA) software (V0.7.17) [17] was employed to align the filtered clean read data with the goat reference genome (ARS1, GCF_001704415.1). SAMtools software (V1.8-20) [18] was then used to convert the resulting sam file into a bam file and to sort the bam file. The MarkDuplicates module within the Genome Analysis Toolkit (GATK) software version 3.8 [19] was employed to eliminate duplicate reads from the sorted bam files. This process led to the generation of the final bam file, which was then subjected to indexing. The HaplotypeCaller module in GATK was utilized to detect SNP variations and generate a vcf file. The gene variants that were detected underwent functional annotation by leveraging the ANNOVAR software (version 2023-06-01) suite [20].

### 2.4. Data Quality Control, Genetic Relationship Analysis Based on the IBS Distance Matrix and G Matrix, and PCA Analysis

Plink software (V1.90) [21] was employed to perform the conversion of vcf files into ped and map files, thereby facilitating subsequent in-depth analyses. SNPs with a genotype detection rate of less than 95% (call rate), SNPs with a minimum allele frequency (MAF) of less than 5%, and SNPs with a *p*-value of less than 10^−6^ (Hardy–Weinberg equilibrium) were excluded from the analysis. The identity by state (IBS) matrix and the G matrix were generated through the utilization of the same software in order to conduct an in-depth analysis of the relationships among the individuals. The “--pca 5” parameter in Plink software was employed to calculate the first five principal components. Since the first five principal components can generally account for a considerable proportion of the variance in the original data, this implies that they capture most of the important information in the original data. By selecting these five principal components, we can significantly reduce the dimensionality of the data while retaining the key genetic variation information, thus improving the analysis efficiency. Finally, R (V3.6.0) [22] was used for plotting to visually present the genetic distance, kinship results, and PCA plot.

### 2.5. Genome-Wide Association Study

The fast Generalized Linear Mixed Model (fastGWA-mlm) implemented within GCTA software version 1.94.0beta was employed to perform an analysis of the associations between SNPs and individual traits, specifically, birth weight or weaning weight [23]. In the process of this analysis, the group stratification and the relationship between individuals are fully considered. The calculation formula is as follows:

y = Xα + Zβ + Wu + e

where y is the population phenotype observation vector; α is a vector of covariates (fixed effects), including the gender, the herd, the birth type, and the first 5 principal components; β is the genotype vector; u is for random effects (kinship matrix); and X, Z, and W are the correlation matrices of α, β, and u, respectively. e is the residual vector.

The significance threshold determined by the Bonferroni correction approach for the GWAS was considered overly stringent. Thus, in the present research, the threshold for genome-wide significant associations was modified to *p* = 1 × 10⁻^6^. The genomic inflation factor (denoted as λ) of the test statistics was computed by making use of the slope of the linear regression between the observed quantiles and the theoretical quantiles in the R statistical software environment (version 3.6.0). Under ideal circumstances, when there are no confounding factors such as population structure and kinship, the observed *p*-values should follow a uniform distribution, and in this case, the inflation factor λ = 1. However, in practical GWASs in goats, due to the presence of factors like population stratification, kinship, and linkage disequilibrium, the test statistics often inflate. That is, there are more significantly associated loci observed than expected, resulting in the inflation factor λ > 1. Generally speaking, when λ is close to 1, it indicates that the GWAS results are less affected by confounding factors, the observed association signals are relatively reliable, and the research findings have a high degree of credibility. The significant single-nucleotide polymorphisms (SNPs) are presented as threshold lines within the Manhattan plot. Both the Manhattan plot and the Quantile–Quantile (Q-Q) plot were created by employing the R CMplot package (https://github.com/YinLiLin/R-CMplot, 20 January 2019).

### 2.6. GO Functional Annotation and KEGG Enrichment Analysis of Candidate Genes

The reference genome of goats (ARS1, GCF_001704415.1) was employed to conduct annotation on a 500-kilobase (kb) region of SNPs that are significantly associated with birth weight and weaning weight in Inner Mongolia cashmere goats. Enrichment analyses of Gene Ontology (GO) and the Kyoto Encyclopedia of Genes and Genomes (KEGG) were conducted using the clusterProfiler software package [24]. Additionally, we discussed the relationship between these genes and birth weight and weaning weight.

### 2.7. Statistical Analysis of Population Genetic Parameters for Significant SNPs

We statistically analyzed the genetic parameters of each SNP significantly associated with birth weight and weaning weight in cashmere goats. We wrote Excel functions to calculate genotype frequency, allele frequency, homozygosity (Ho), heterozygosity (He), polymorphism information content (PIC), and effective allele number (Ne). Additionally, we applied the Chi-squared test to determine whether the population was in Hardy–Weinberg equilibrium (*p* > 0.05).

### 2.8. Construction of Haplotype Block and Analysis of Linkage Disequilibrium

Haploview (V4.2) software [25] was utilized to analyze the linkage disequilibrium of SNPs that conformed to Hardy–Weinberg equilibrium and to construct haplotype blocks. The D′ and r^2^ values were employed to assess the linkage disequilibrium between the two SNPs. Generally, D′ > 0.33 and r^2^ > 0.1 indicate meaningful linkage disequilibrium, while D′ > 0.8 and r^2^ > 0.33 signify strong linkage disequilibrium. Furthermore, higher values of D′ and r^2^ reflect a stronger linkage disequilibrium relationship between SNPs.

### 2.9. Association Analysis of Haplotype Combination with Birth Weight and Weaning Weight Traits

One-way analysis of variance (ANOVA) was carried out by means of SAS (V9.2) software [26]. In cases where only two genotypes were detected at a specific locus, the *t*-test was utilized. Moreover, the association analysis between phenotypes and genotypes was conducted using SAS (V9.2) software. The model of correlation analysis between haplotype combination and birth weight (or weaning weight) is as follows:

Y_ijdk_ = μ + H_i_ + F_j_ + S_d_ + T_k_ + E_ijdk_

where μ is the population average (birth weight, weaning weight), H_i_ is the haplotype effect, F_j_ is the herd, S_d_ is the gender, T_k_ is the birth type, and E_ijdk_ is random error. Duncan’s multiple range test was employed to evaluate the significance of the differences among genotypes (*p* < 0.05), and the data were presented in the form of “mean ± standard error of the mean”.

## 3. Results

### 3.1. Descriptive Statistics of Phenotypic Data

The descriptive statistical outcomes of the phenotypic data regarding the traits of birth weight and weaning weight are shown in Table 1. As indicated by Table 1, the mean birth weight of single-offspring Inner Mongolia cashmere goats is the highest, amounting to 2.71 kg, with a standard deviation of 0.44. The mean weaning weight of single-offspring Inner Mongolia cashmere goats is also the highest, reaching 15.98 kg, with a standard deviation of 2.59. This implies that the degree of dispersion within the experimental population is relatively low, suggesting that single-offspring individuals have a better growth and development status compared to multi-offspring individuals.

**Figure 2:**
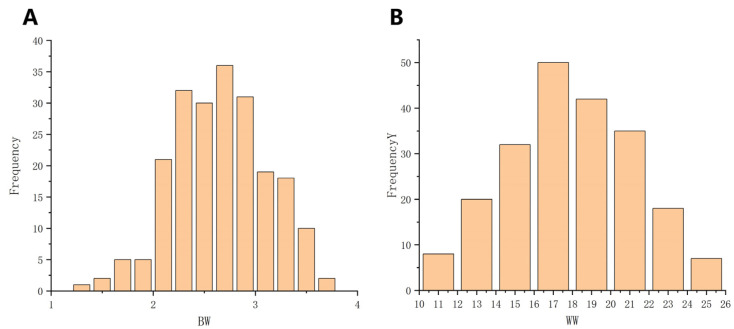
Phenotypic frequency distribution of birth weight and weaning weight. (**A**) Birth weight trait; (**B**) weaning weight trait.

Moreover, the coefficient of variation of the birth weight for single-offspring individuals is 16.18%; the coefficient of variation of the weaning weight for single-offspring individuals is 16.22%; and the coefficient of variation of the weaning weight for twin-offspring individuals is 17.16%. All coefficients of variation are greater than 15%, representing moderate variation, which indicates that through selection breeding for birth weight and weaning weight, significant genetic progress might be achieved in a relatively short time. Subsequently, Origin software (V2022) was employed to conduct a normality test on the phenotypic values, confirming that all traits adhered to a normal distribution (Figure 2), thus allowing for the subsequent genome-wide association analysis.

### 3.2. Adjustment for Fixed Effects

We utilized a linear mixed model to account for fixed effects, specifically, non-genetic factors. As indicated in Tables S1 and S2, both gender and birth type significantly influenced birth weight (*p* < 0.01). Additionally, herd also had a significant impact on birth weight (*p* < 0.05). Furthermore, gender, herd, and birth type were all found to significantly affect weaning weight traits (*p* < 0.01), whereas birth year did not demonstrate a significant effect on either birth weight or weaning weight (*p* > 0.05). Consequently, this study excluded birth year as a variable and incorporated gender, herd, and birth type as fixed effects in the GWAS model for both birth weight and weaning weight.

### 3.3. Data Quality Control, Genetic Relationship, and PCA Analysis

**Figure 3:**
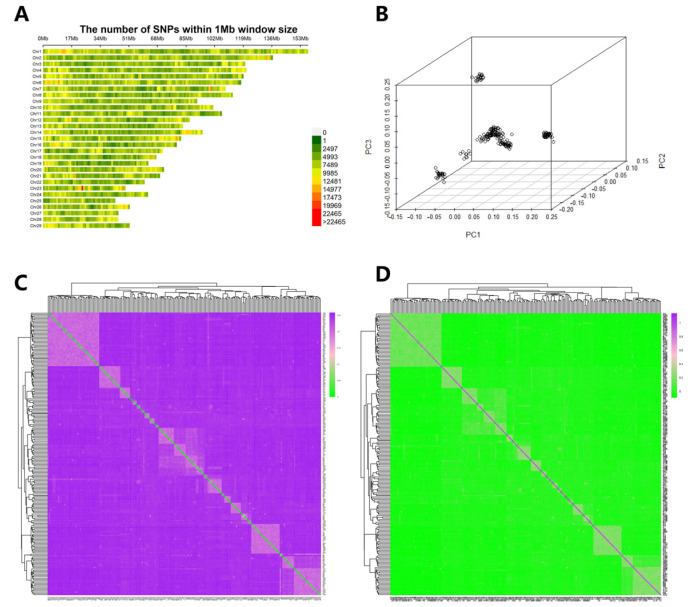
Data quality control, PCA, and genetic relationship analysis. (**A**) Map of the distribution of SNPs on chromosomes; (**B**) Diagram of principal component analysis; (**C**) IBS genetic distance visualization; (**D**) G matrix visualization.

Plink software was utilized to perform quality control on 34,248,064 SNPs, resulting in the exclusion of 460,822 SNPs with a detection rate of less than 95% (--geno 0.05). The residual SNPs were subjected to a filtering process based on the minimum allele frequency criterion, Hardy–Weinberg equilibrium principle, and individual detection rate parameters (with a minimum allele frequency threshold set at 0.05, a Hardy–Weinberg equilibrium significance level of 1 × 10^−6^, and an individual detection rate cutoff of 0.1). This filtering procedure resulted in a total of 17,258,261 SNPs. These SNPs were evenly distributed across the 29 autosomal pairs (Figure 3A), and no regions were left uncovered, thereby allowing for subsequent GWAS analysis.

To prevent false-positive associations resulting from individual kinship and population stratification, we first analyzed the population structure of 212 Inner Mongolia cashmere goats. As illustrated in Figure 3B, our analysis reveals that there is significant population stratification within the test sample, with individuals broadly categorized into five distinct groups. In this study, the first five principal components were selected as covariates to be incorporated into the mixed linear model, based on insights from prior research, in order to correct for the effects of population stratification.

The genetic distance derived from IBS and G-matrix facilitates phylogenetic analysis of populations even in the absence of genealogical or progenitor samples. Findings revealed that the IBS (identity by state) distance values within the Inner Mongolia cashmere goat population spanned from 0.1301 to 0.2575, with an average genetic distance registering at 0.2392 ± 0.0135. This implies that the average genetic distance among individuals in the Inner Mongolia cashmere goat population is relatively large, signifying a significant degree of variability. The visual representations of the IBS genetic distance matrix and the G-matrix affinity for the entire population are depicted in Figure 3C,D. Most individuals were found to be distantly related, while some cashmere goats exhibited closer relationships due to the presence of family groups among the sequenced individuals.

### 3.4. GWAS for Birth Weight and Weaning Weight Traits

#### 3.4.1. Birth Weight Trait

**Figure 4:**
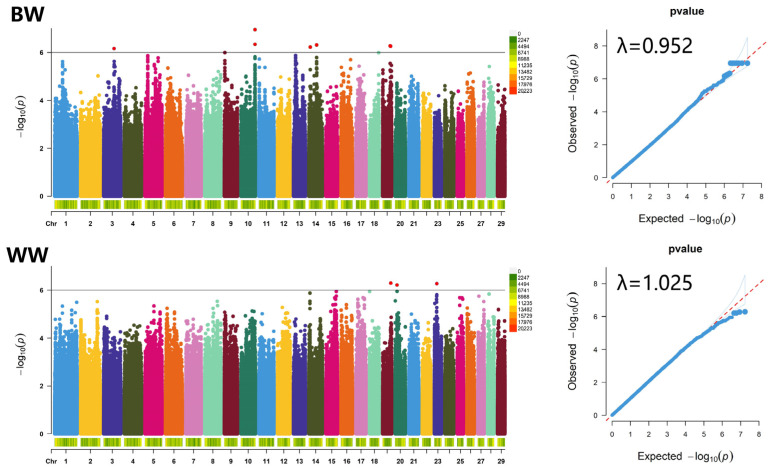
Manhattan (**left**) and Q-Q (**right**) plots of birth weight and weaning weight traits in Inner Mongolia cashmere goats. Genome-wide significant SNPs are shown in red. The blue thin lines in the Q-Q plot represent the confidence intervals of the data points. BW, birth weight; WW, weaning weight.

Utilizing the resequencing data obtained from 212 Inner Mongolia cashmere goats of the Erlangshan type, we carried out a GWAS to explore birth weight traits. Using a correction threshold of *p* < 1 × 10^−6^, we identified 17 significantly associated SNPs located on chromosomes 3, 10, 14, and 19 (Table 2, Figure 4). The Q-Q plot revealed a genomic inflation factor (λ) of 0.952, suggesting that there was no significant inflation in the genome (0.95–1.05), thereby affirming the credibility of the results.

Nine SNPs (SNP4-SNP12) on chromosome 10 were annotated to the intron region of the *KCNN2* gene. Chr14_g.54137103A>G on chromosome 14 was annotated in the intron region of *NKAIN3* gene. Chr19_g.52517379T>C and chr19_g.55261375C>T on chromosome 19 were annotated to the intron region of the *RBFOX3* and *CASKIN2* genes, respectively (Table 2). Among the 17 SNPs that were significantly associated, the SNP that explained the phenotypic variation the most was chr14_g.54137103A>G, which could explain 2.93% of the phenotypic variation, corresponding to a *p*-value of 4.85 × 10^−7^ and an effect value of −0.1880. The SNP with the smallest explained phenotypic variation was chr10_g.98206421T>C, which could explain 2.39% of the phenotypic variation, with a *p*-value of 4.58 × 10^−7^ and a corresponding effect value of 0.3890 (Table 2).

#### 3.4.2. Weaning Weight Trait

Four SNPs significantly associated with weaning weight were identified, located on chromosomes 19, 20, and 23, respectively (Table 2, Figure 4). The Q-Q plot indicates a genome inflation factor (λ) of 1.025, suggesting no significant genome inflation (0.95–1.05), thereby supporting the credibility of the results.

The variant chr19_g.56428712A>T on chromosome 19 is located within the intron region of the *TTYH2* gene. Similarly, chr23_g.16477721T>G on chromosome 23 is situated in the intron region of the *FARS2* gene (Table 2). Among the four significantly associated SNPs, chr19_g.56428712A>T accounted for the largest proportion of phenotypic variation, explaining 2.58% of the variation, with a *p*-value of 5.09 × 10^−7^ and an effect size of −1.1205. In contrast, chr23_g.16477721T>G exhibited the smallest explained phenotypic variation at 2.45%, with a *p*-value of 5.33 × 10^−7^ and an effect size of 1.0185 (Table 2).

### 3.5. Annotation and Enrichment Analysis of Candidate Genes

**Figure 5:**
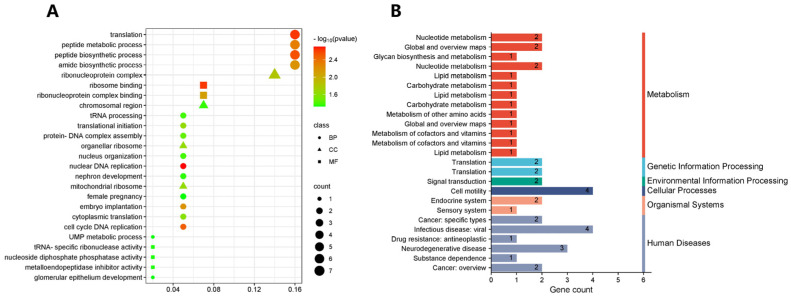
Bar graph presenting the results of partial Gene Ontology (GO) and Kyoto Encyclopedia of Genes and Genomes (KEGG) enrichment analysis of candidate genes. (**A**) GO enrichment outcomes. In the legend, BP stands for biological process, CC denotes cellular component, and MF represents molecular function. (**B**) KEGG enrichment results.

We annotated 21 SNPs that were significantly associated with birth weight and weaning weight in Inner Mongolia cashmere goats, leading to the identification of 117 candidate genes. We subsequently employed the clusterProfiler (V4.0) software package to analyze the GO function and KEGG signaling pathway enrichment of these 117 candidate genes. As elaborated in Tables S4 and S5, these genes exhibited significant enrichment in 1004 GO terms and 123 KEGG pathways. Partial GO and KEGG enrichment analysis results for candidate genes are visualized in Figure 5.

According to the literature, the genes *RUNX1T1*, *C1QTNF1*, *TIMP2*, *GALR2*, *GALK1*, *ATP5H*, *ITGB4*, *GRB2*, *GGA3*, *NT5C*, *MYO15B*, and *ERBIN* are closely associated with biological processes such as skeletal muscle formation, embryonic development, and intramyocellular fat deposition. In our study, *RUNX1T1* is found to be enriched in two molecular functions: transcription corepressor activity and transcription coregulator activity, as well as in the KEGG signaling pathways related to acute myeloid leukemia and transcriptional misregulation in cancer. *C1QTNF1* is enriched in biological processes such as cell activation and the positive regulation of transport. *TIMP2* is associated with biological processes including the regulation of proteolysis, the negative regulation of molecular function, and peptidase regulator activity, along with zinc ion binding and other molecular functions. Additionally, *GALR2* is enriched in KEGG signaling pathways related to signaling molecules and interactions.

*GALK1* is enriched in biological processes such as carbohydrate catabolic processes and the glycolytic process, as well as in KEGG signaling pathways, including galactose metabolism. *ITGB4* is enriched in KEGG signaling pathways such as ECM–receptor interaction and the PI3K-Akt signaling pathway. *GRB2* is associated with biological processes including response to insulin and cellular import, as well as cellular components such as the nucleolus and cell–cell junctions. Its molecular functions include ephrin receptor binding and protein tyrosine kinase binding, alongside KEGG signaling pathways such as JAK-STAT, ErbB, Ras, and MAPK. *GGA3* is enriched in cellular components such as early endosomes and intracellular protein transport, with molecular functions including phosphatidylinositol binding and phospholipid binding, and is also involved in the KEGG signaling pathway associated with lysosomes. *NT5C* is enriched in biological processes such as purine deoxyribonucleotide metabolic processes and ribonucleotide catabolic processes, with the molecular function of phosphatase activity, and is linked to KEGG signaling pathways such as pyrimidine metabolism and purine metabolism. *MYO15B* is enriched in motor protein signaling pathways. *ERBIN* is involved in biological processes such as the negative regulation of NF-kappaB transcription factor activity and protein targeting, with cellular components including nuclear specks and the basal part of the cell, and is associated with the KEGG signaling pathway related to NOD-like receptor signaling. Notably, *ATP5H* and *FAM65B* were not enriched in GO terms or KEGG signaling pathways. Although some of these GO terms and KEGG signaling pathways do not directly contribute to growth, they play mutually reinforcing roles within the broader context of the organism’s environment.

### 3.6. Population Genetic Parameter Statistics

The statistics of genetic parameters for the cashmere goat population are presented in Table 3. Among the 17 SNPs significantly associated with birth weight, 6 SNPs exhibited three genotypes, while the remaining 11 SNPs displayed two genotypes. Notably, SNPs 4, 5, 6, 8, 9, 10, 11, and 12 demonstrated the lowest heterozygosity at 0.098, whereas SNP15 exhibited the highest heterozygosity of 0.497. The polymorphism information content (PIC) for SNP15 indicated moderate polymorphism (0.25 < PIC < 0.50), while the other 16 SNPs showed low polymorphism (0 < PIC < 0.25). This suggests that the degree of genetic variation among the significantly associated SNPs in this population is limited, indicating a tendency towards stability under prolonged natural selection and artificial breeding. The chi-squared test confirmed that all 17 SNPs conformed to the Hardy–Weinberg equilibrium (*p* > 0.05), allowing for their use in further analysis.

Among the four SNPs significantly associated with weaning weight, three genotypes were identified in SNP18 and SNP21, while two genotypes were present in SNP19 and SNP20. The lowest heterozygosity was observed in SNP19 and SNP20, at 0.131, whereas the highest heterozygosity was found in SNP21, at 0.389. The count of effective alleles within the Inner Mongolia cashmere goat population spanned from 1.151 to 1.636. The Polymorphism Information Content (PIC) values of SNP19 and SNP20 suggested low-level polymorphism (0 < PIC < 0.25), whereas SNP18 and SNP21 displayed moderate-level polymorphism (0.25 < PIC < 0.50). The Chi-squared test revealed that the four SNPs were in accordance with the Hardy–Weinberg Equilibrium (HWE) (*p* > 0.05), thereby enabling subsequent analyses (Table 3). Additionally, the number of effective alleles in the Inner Mongolia cashmere goat population based on 21 SNPs significantly associated with birth weight and weaning weight ranged from 1.109 to 1.990, which also reflected the high genetic diversity of the Inner Mongolia cashmere goat population.

### 3.7. Haplotype Analysis

**Figure 6:**
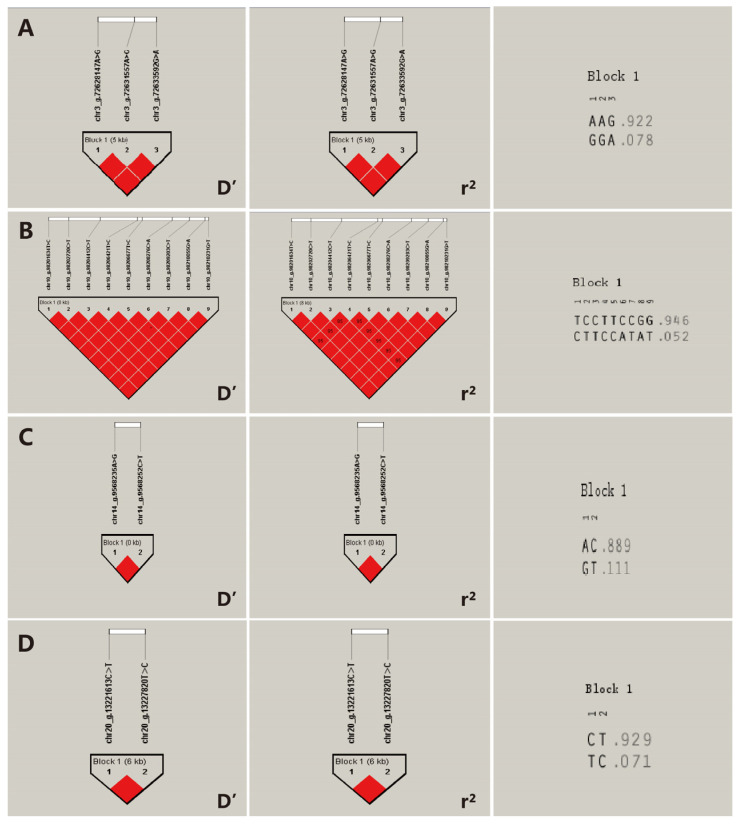
Findings from the linkage disequilibrium analysis and haplotype determination of SNPs that are significantly associated with birth weight and weaning weight in IMCGs. (**A**) The D’ value, r^2^ value, and haplotype frequency of the linkage block constituted by three SNPs within the *RWDD3* gene. (**B**) The D’ value, r^2^ value, and haplotype frequency of the linkage block formed by nine SNPs in the *KCNN2* gene. (**C**) The D’ value, r^2^ value, and haplotype frequency of the linkage block composed of two SNPs in the *RUNX1T1* gene. (**D**) The D’ value, r^2^ value, and haplotype frequency of the linkage block formed by two SNPs in the *LOC102178014* gene. The linkage relationship between loci was evaluated based on D’ and r^2^ values. Specifically, when D’ = 1 or r^2^ = 1, it is referred to as complete linkage; when D’ = 0 or r^2^ = 0, it indicates no linkage or linkage equilibrium; and when D’ > 0.80 and r^2^ > 0.33, it implies strong linkage. The numbers on the right represent the probabilities corresponding to the respective haplotype combinations.

The Haploview (V4.2) software was employed to conduct a linkage disequilibrium analysis on the SNPs that were significantly associated with the birth weight and weaning weight traits in Inner Mongolia cashmere goats and that conformed to the Hardy–Weinberg equilibrium. SNPs significantly correlated with the birth weight of Inner Mongolia cashmere goats, forming three distinct blocks (Figure 6). Notably, chr2_g.8302494A>G and chr2_g.8303830C>T in the *RWDD3* gene were completely linked with chr2_g.8303941T>C (D′ = 1). Additionally, the nine SNPs (SNP4-SNP12) of the *KCNN2* gene exhibited complete linkage (D′ = 1). The *RUNX1T1* gene, specifically chr14_g.9568235A>G, was fully linked to chr14_g.9568252C>T (D′ = 1). Furthermore, SNPs significantly associated with weaning weight traits in Inner Mongolia cashmere goats formed a single block (Figure 6), with chr20_g.13221613C>T and chr20_g.13227820T>C in the LOC102178014 gene being completely linked (D′ = 1).

Through the computation of haplotype combinations and their corresponding frequencies, it was demonstrated that within the Inner Mongolia cashmere goat population, the three SNPs that are significantly associated with the birth weight of Inner Mongolia cashmere goats, respectively, constituted two, three, and two haplotype combinations. The theoretical number of haplotype combinations was 2^3^ (8), 2^9^ (512), and 2^2^ (4) species. The haplotype combinations in the three haplotype blocks were designated as A1~A2, B1~B3, and C1~C2, respectively. For specific haplotype combination frequencies, refer to Table 4. Additionally, one haplotype block significantly associated with weaning weight traits in Inner Mongolia cashmere goats formed two haplotype combinations within the population, with a theoretical haplotype number of 2^2^ (4) species. These two haplotype combinations were labeled D1~D2, and their specific frequencies are presented in Table 4.

### 3.8. Association Analysis of Haplotype Combinations with Birth Weight and Weaning Weight Traits

**Figure 7:**
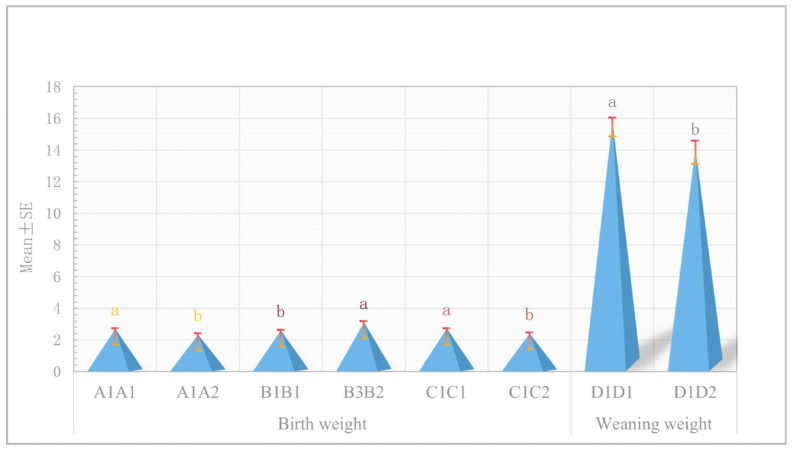
Correlation analysis was carried out to explore the relationships between haplotype combinations and phenotypic traits of birth weight and weaning weight in IMCGs. Distinct letters denote significant differences, while identical letters signify non-significant differences.

To further investigate the potential effects of haplotype combinations on phenotypes, we conducted a correlation analysis between the generated haplotype combinations and the phenotypes of birth weight and weaning weight (Table 5, Figure 7). Samples with an individual count of less than three were excluded from the multiple-comparison analysis. The results indicated that the birth weight of haplotype combination A1A1 was significantly greater than that of A1A2 (*p* < 0.05). Similarly, the birth weight of haplotype combination B3B2 was significantly higher than that of B1B1 (*p* < 0.05), and the birth weight of haplotype combination C1C1 was significantly greater than that of C1C2 (*p* < 0.05). Furthermore, the weaning weight of haplotype combination D1D1 was significantly higher than that of D1D2 (*p* < 0.05).

## 4. Discussion

Non-genetic factors (fixed effects) and population stratification are critical elements influencing the accuracy of GWAS results. Notably, population stratification can induce false associations between SNPs and target traits, resulting in false positives that arise from a lack of true association between SNPs or candidate genes and the studied traits [27]. Variance analysis reveals that gender, herd, and birth type significantly affect birth weight and weaning weight. To mitigate the non-genetic effects attributable to these factors on the target traits, they are incorporated as fixed effects into the mixed linear model for correction. Furthermore, Plink (V1.90) software was utilized for principal component analysis, which indicated a clear presence of population stratification. To address the potential confounding influence of this phenomenon on GWAS results, PCA results were integrated into the mixed linear model as covariates to enhance the accuracy and reliability of the findings. Finally, the accuracy of the GWAS results was assessed based on the congruence between expected and observed values, as well as the λ value in the Q-Q-plot. After correcting for non-genetic factors and population stratification, it was found that the graph fitted well, with a λ value falling within the acceptable range of 0.95 to 1.05. This suggests that our model is appropriately configured, with some sites exhibiting an upward tilt, indicating a substantial effect caused by these SNPs. In summary, through variance analysis, principal component analysis, and mixed linear model correction, the incidence of false positives in GWAS results was effectively reduced, thereby enhancing the accuracy. This provides a solid foundation for the further exploration of genetic characteristics and breeding in cashmere goats.

We identified 21 significant SNPs associated with birth weight and weaning weight through GWASs, resulting in a total of 117 significant genes being identified via gene annotation. Among these, *RUNX1T1*, a protein-coding gene, interacts with *RUNX1*, a transcription factor located in the nucleus, to jointly regulate gene expression and the process of cell differentiation. In the liver, *RUNX1T1* has been shown to improve the body fat index in obese mice following endurance exercise. Additionally, other studies have indicated that miR-27a, miR-146a-5p, and miR-221-3p can target *RUNX1T1* to regulate cell proliferation [28,29,30]. *C1QTNF1* exhibited a trend of differential expression in human smooth muscle cells and was found to accelerate cardiac hypertrophy and fibrosis in cardiomyocytes [31,32]. The expression of *TIMP2* was elevated in the skeletal muscle of canines with X-linked muscular dystrophy in Japan. Muscle satellite cells are crucial in muscle homeostasis and regeneration, and the targeted combination of *TIMP2* and PAM can promote the aging of muscle satellite cells [33,34]. *GALR2* has potential as a therapeutic target in treating insulin resistance, as its activation alleviates insulin resistance through the P38MAPK/PGC-1α/GLUT4 and AKT/AS160/GLUT4 pathways in mouse skeletal muscle. Furthermore, *GALR2* and *GALR3*, which serve as receptors for *SPX*, play significant roles in regulating lipid and carbohydrate metabolism in both human and animal adipose tissues [35,36,37]. Some researchers have identified the *GMDS*, *GALK1*, and *ITGB4* genes in Dolper and Hu sheep through whole-genome resequencing technology, which are significantly correlated with immune function. Given that immune function plays a crucial role in the growth of domestic animals, this discovery offers a new perspective on our understanding of domestic animal growth [38]. The proteins in bovine skeletal muscle were monitored for variations in intramuscular fat content, revealing an upregulation trend for *ATP5H* [39]. In a genome-wide association study on chickens, *GGA3* was found to be significantly correlated with body composition and meat quality traits [40]. Following the whole-genome bisulfite sequencing and transcriptome sequencing of skeletal muscle from ducks at day 21 (E21) and day 28 (E28), the *ERBIN* gene was identified as playing a key role in regulating skeletal muscle development [41]. An epigenome-wide association study demonstrated that the percentage of very-low-birth-weight infants who were breastfed was significantly associated with the DNA methylation levels of two specific CpGs-cg03744440 (located on the *MYO15B* gene) and cg00851389 (located on *metallothionein 1A*) at 5.5 years of age [42]. A study on tissue expression profiles indicated that *NT5C1A* and *NT5C2* are expressed in skeletal muscle [43]. *GRB2* is a necessary factor for the growth and transformation of mouse embryonic cells [44]. While disruption to *GRB2* binding does not directly affect embryonic development, it results in significant muscle reduction in the extremities, accompanied by a general loss of secondary fibers [45]. Therefore, it is speculated that these genes may be closely related to the early growth traits of Inner Mongolia cashmere goats and could be utilized to enhance the selection and breeding of growth traits.

Haplotypes can provide more comprehensive information than SNPs, as they account for non-allelic interactions and linkage imbalances among multiple mutation sites [46,47]. In this study, four haplotype blocks were constructed, with the first three haplotype blocks influencing birth weight traits, encompassing 14 SNPs located within the *RWDD3*, *KCNN2*, and *RUNX1T1* genes. There is complete linkage among the SNPs within each haplotype block. The frequency of haplotype combination A1 (AAG) in the *RWDD3* gene was as high as 0.922, while the frequency of haplotype combination B1 (TCCTTCCGG) in the *KCNN2* gene reached 0.946, and the frequency of haplotype combination C1 (AC) in the *RUNX1T1* gene was 0.889. These haplotype combinations are highly likely to become fixed within the population. Individuals with the B3B2 haplotype combination exhibited superior phenotypic values compared to those with other haplotype combinations (A1A1, A1A2, B1B1, C1C1, C1C2), although the number of individuals with B3B2 was limited. In conclusion, stable homozygous haplotype combinations such as A1A1 (AAAAGG), C1C1 (AACC), and D1D1 (CCTT), which are associated with high phenotypic values, can be utilized for the genetic improvement of birth weight and weaning weight in Inner Mongolia cashmere goats.

## 5. Conclusions

Using genome resequencing data, we identified 21 SNPs significantly associated with birth weight and weaning weight traits through genome-wide association analysis and haplotype analysis. Notable candidate genes include *RUNX1T1*, *ERBIN*, *MYO15B*, *NT5C*, *GRB2*, *ITGB4*, and *GALK*. We constructed the homozygous haplotype combinations A1A1, C1C1, and D1D1. The SNPs and haplotypes identified in this study provide candidate genetic variants for molecular marker-assisted breeding related to the early growth traits of Inner Mongolia cashmere goats, thereby enhancing production performance and economic benefits in breeding.

## Figures and Tables

**Table 1:** Descriptive statistics for birth weight and weaning weight.

Traits	Birth Type	Dam Age	Number	Mean	Max	Min	SD	CV (%)
Birth weight	Singletons	3	183	2.71	3.73	1.38	0.44	16.18
	Twins	3	27	2.23	2.87	1.7	0.32	14.33
	Triplets	3	2	2.00	2.04	1.95	0.06	3.12
Weaning weight	Singletons	3	183	15.98	24.53	8.69	2.59	16.22
	Twins	3	27	13.43	19.23	9.86	2.30	17.16
	Triplets	3	2	12.36	14.81	9.91	3.46	28.02

**Table 2:** Significant SNPs and candidate genes associated with birth weight and weaning weight traits.

Trait	SNP_Number	SNP	BETA	*p*-Value	r^2^ (%)	Distance (bp)	Structure_Type	Gene
Birth weight	SNP1	chr3_g.72628147A>G	−0.3449	6.81 × 10^−7^	2.66	−361,498	intergenic	*RWDD3*
	SNP2	chr3_g.72631557A>G	−0.3449	6.81 × 10^−7^	2.66	−364,908	intergenic	*RWDD3*
	SNP3	chr3_g.72633592G>A	−0.3449	6.81 × 10^−7^	2.66	−366,943	intergenic	*RWDD3*
	SNP4	chr10_g.98201634T>C	0.4349	1.11 × 10^−7^	2.65	within	intronic	*KCNN2*
	SNP5	chr10_g.98202720C>T	0.4349	1.11 × 10^−7^	2.65	within	intronic	*KCNN2*
	SNP6	chr10_g.98204412C>T	0.4349	1.11 × 10^−7^	2.65	within	intronic	*KCNN2*
	SNP7	chr10_g.98206421T>C	0.3890	4.58 × 10^−7^	2.39	within	intronic	*KCNN2*
	SNP8	chr10_g.98206677T>C	0.4349	1.11 × 10^−7^	2.65	within	intronic	*KCNN2*
	SNP9	chr10_g.98208276C>A	0.4349	1.11 × 10^−7^	2.65	within	intronic	*KCNN2*
	SNP10	chr10_g.98209203C>T	0.4349	1.11 × 10^−7^	2.65	within	intronic	*KCNN2*
	SNP11	chr10_g.98210055G>A	0.4349	1.11 × 10^−7^	2.65	within	intronic	*KCNN2*
	SNP12	chr10_g.98210231G>T	0.4349	1.11 × 10^−7^	2.65	within	intronic	*KCNN2*
	SNP13	chr14_g.9568235A>G	−0.2871	5.96 × 10^−7^	2.73	228,432	intergenic	*RUNX1T1*
	SNP14	chr14_g.9568252C>T	−0.2871	5.96 × 10^−7^	2.73	228,415	intergenic	*RUNX1T1*
	SNP15	chr14_g.54137103A>G	−0.1880	4.85 × 10^−7^	2.93	within	intronic	*NKAIN3*
	SNP16	chr19_g.52517379T>C	−0.2512	5.31 × 10^−7^	2.79	within	intronic	*RBFOX3*
	SNP17	chr19_g.55261375C>T	−0.2657	5.44 × 10^−7^	2.77	within	intronic	*CASKIN2*
Weaning weight	SNP18	chr19_56428712A>T	−1.1205	5.09 × 10^−7^	2.58	within	intronic	*TTYH2*
	SNP19	chr20_13221613C>T	−1.8399	6.11 × 10^−7^	2.52	−177,193	intergenic	*LOC102178014*
	SNP20	chr20_13227820T>C	−1.8399	6.11 × 10^−7^	2.52	−183,400	intergenic	*LOC102178014*
	SNP21	chr23_16477721T>G	1.0185	5.33 × 10^−7^	2.45	within	intronic	*FARS2*

Note: r^2^ (%) denotes the proportion of phenotypic variance accounted for by the SNP. Positive values in the “Distance” column signify the distance between the SNP and the upstream region of the gene; negative values in the “Distance” column represent the distance between the SNP and the downstream region of the gene. The term “Within” indicates that the SNP is situated within the gene. In this table, only genes where the SNP is located within the gene or the genes closest to the SNP are presented as potential candidate genes. The comprehensive results of gene annotation are presented in Table S3.

**Table 3:** Population genetic parameters of SNPs and potential candidate genes associated with birth weight and weaning weight traits in Inner Mongolia cashmere goats.

Trait	SNP_Number	Allelic Frequency		HWE		Ho	He	PIC	Ne
		Major	Minor	χ^2^	*p*				
Birth weight	SNP1	0.922	0.078	1.51	0.219	0.856	0.144	0.133	1.168
	SNP2	0.922	0.078	1.51	0.219	0.856	0.144	0.133	1.168
	SNP3	0.922	0.078	1.51	0.219	0.856	0.144	0.133	1.168
	SNP4	0.948	0.052	0.635	0.426	0.902	0.098	0.094	1.109
	SNP5	0.948	0.052	0.635	0.426	0.902	0.098	0.094	1.109
	SNP6	0.948	0.052	0.635	0.426	0.902	0.098	0.094	1.109
	SNP7	0.946	0.054	0.254	0.615	0.897	0.103	0.097	1.114
	SNP8	0.948	0.052	0.635	0.426	0.902	0.098	0.094	1.109
	SNP9	0.948	0.052	0.635	0.426	0.902	0.098	0.094	1.109
	SNP10	0.948	0.052	0.635	0.426	0.902	0.098	0.094	1.109
	SNP11	0.948	0.052	0.635	0.426	0.902	0.098	0.094	1.109
	SNP12	0.948	0.052	0.635	0.426	0.902	0.098	0.094	1.109
	SNP13	0.889	0.111	0.178	0.673	0.803	0.197	0.178	1.246
	SNP14	0.889	0.111	0.178	0.673	0.803	0.197	0.178	1.246
	SNP15	0.535	0.465	1.731	0.188	0.503	0.497	0.374	1.99
	SNP16	0.856	0.144	0.116	0.733	0.754	0.246	0.216	1.327
	SNP17	0.874	0.126	0.181	0.67	0.78	0.22	0.196	1.281
Weaning weight	SNP18	0.778	0.222	0.319	0.572	0.655	0.345	0.286	1.527
	SNP19	0.929	0.071	1.229	0.268	0.869	0.131	0.123	1.151
	SNP20	0.929	0.071	1.229	0.268	0.869	0.131	0.123	1.151
	SNP21	0.736	0.264	0.182	0.67	0.611	0.389	0.313	1.636

Note: SNP, single-nucleotide polymorphism; HWE, Hardy–Weinberg equilibrium test; Ho, homozygosity; He, heterozygote; PIC, polymorphism information content; Ne, effective allele number. The chi-square statistic (χ^2^) is a measure that quantifies the discrepancy between the observed frequencies and the expected frequencies during a chi-square test. The *p* in a chi—square test is the probability of obtaining a test statistic as extreme as, or more extreme than, the observed χ^2^, assuming that the null hypothesis is true.

**Table 4:** Haplotype combinations and frequencies for birth weight and weaning weight in IMCGs.

Trait	Gene	Tag	Haplotype	Frequency
Birth weight	*RWDD3*	A1	AAG	0.922
		A2	GGA	0.078
	*KCNN2*	B1	TCCTTCCGG	0.946
		B2	CTTCCATAT	0.052
		B3	TCCCTCCGG	0.002
	*RUNX1T1*	C1	AC	0.889
		C2	GT	0.111
Weaning weight	*LOC102178014*	D1	CT	0.929
		D2	TC	0.071

**Table 5:** Association analysis of haplotype combination with birth weight and weaning weight traits in IMCGs.

Trait	Gene	Haplotype Combination	Number	Mean ± SE
Birth weight	*RWDD3*	AAAAGG (A1A1)	179	2.6989 ± 0.0328 ^a^
		AGAGGA (A1A2)	33	2.3321 ± 0.0733 ^b^
	*KCNN2*	TTCCCCTTTTCCCCGGGG (B1B1)	190	2.5937 ± 0.0316 ^b^
		TCCTCTTCTCCACTGAGT (B3B2)	21	3.1 ± 0.0821 ^a^
	*RUNX1T1*	AACC (C1C1)	167	2.7072 ± 0.0350 ^a^
		AGCT (C1C2)	43	2.4119 ± 0.0583 ^b^
Weaning weight	*LOC102178014*	CCTT (D1D1)	182	15.8704 ± 0.1973 ^a^
		CTTC (D1D2)	30	14.1227 ± 0.4646 ^b^

Note: The same letter indicates no significant difference, while different letters indicate significant differences.

## Data Availability

The supporting data of this study are available from the corresponding authors upon request.

## Supplementary Materials

The following are available online at https://www.mdpi.com/article/10.3390/vetsci12030192/s1: Table S1: Effect of gender, herd, lambing year (2020, 2021), and birth type on birth weight in IMCGs; Table S2: Effect of gender, herd, lambing year (2020, 2021), and birth type on weaning weight in IMCGs; Table S3: Annotation results for significantly associated SNPs of birth weight and weaning weight traits in IMCGs; Table S4: GO enrichment analysis results for candidate genes for birth weight and weaning weight traits in IMCGs; Table S5: KEGG enrichment analysis results for candidate genes for birth weight and weaning weight traits in IMCGs.

## Abbreviations

WW	weaning weight
BW	birth weight
GWAS	genome-wide associated study
SNPs	single-nucleotide polymorphisms
LD	linkage disequilibrium
IMCGs	Inner Mongolian cashmere goats
HWE	Hardy–Weinberg equilibrium test
Ho	homozygosity
He	heterozygosity
PIC	polymorphism information content
Ne	effective allele number
MAF	minor allele frequency
IBS	identity by state

## References

[1] Zhao Q., Huang C., Chen Q., Su Y., Zhang Y., Wang R., Su R., Xu H., Liu S., Ma Y. (2024). Genomic Inbreeding and Runs of Homozygosity Analysis of Cashmere Goat. Animals.

[2] Wu Z., Hai E., Di Z., Ma R., Shang F., Wang M., Liang L., Rong Y., Pan J., Su R. (2021). Chi-miR-130b-3p regulates Inner Mongolia cashmere goat skin hair follicles in fetuses by targeting Wnt family member 10A. G3.

[3] Han W., Yang F., Wu Z., Guo F., Zhang J., Hai E., Shang F., Su R., Wang R., Wang Z. (2020). Inner Mongolian Cashmere Goat Secondary Follicle Development Regulation Research Based on mRNA-miRNA Co-analysis. Sci. Rep..

[4] Wang Y., Tan H., Zhang M., Zhao R., Wang S., Qin Q., Wang J., Zhang C., Tao M., Ma M. (2020). The Hybrid Genome of a New Goldfish-Like Fish Lineage Provides Insights Into the Origin of the Goldfish. Front. Genet..

[5] Zhuang Z., Xu L., Yang J., Gao H., Zhang L., Gao X., Li J., Zhu B. (2020). Weighted Single-Step Genome-Wide Association Study for Growth Traits in Chinese Simmental Beef Cattle. Genes.

[6] Tam V., Patel N., Turcotte M., Bossé Y., Paré G., Meyre D. (2019). Benefits and limitations of genome-wide association studies. Nat. Rev. Genet..

[7] Zhang S., Zhao H., Lei C., Pan C., Chen H., Lin Q., Lan X. (2020). Effects of genetic variations within goat PITX2 gene on growth traits and mRNA expression. Anim. Biotechnol..

[8] Wang Z., Wang C., Guo Y., She S., Wang B., Jiang Y., Bai Y., Song X., Li L., Shi L. (2020). Screening of Deletion Variants within the Goat PRDM6 Gene and Its Effects on Growth Traits. Animals.

[9] Zhang L., Wang F., Gao G., Yan X., Liu H., Liu Z., Wang Z., He L., Lv Q., Wang Z. (2021). Genome-Wide Association Study of Body Weight Traits in Inner Mongolia Cashmere Goats. Front. Vet. Sci..

[10] Gu B., Sun R., Fang X., Zhang J., Zhao Z., Huang D., Zhao Y., Zhao Y. (2022). Genome-Wide Association Study of Body Conformation Traits by Whole Genome Sequencing in Dazu Black Goats. Animals.

[11] Easa A.A., Selionova M., Aibazov M., Mamontova T., Sermyagin A., Belous A., Abdelmanova A., Deniskova T., Zinovieva N. (2022). Identification of Genomic Regions and Candidate Genes Associated with Body Weight and Body Conformation Traits in Karachai Goats. Genes.

[12] Schafer A.J., Hawkins J.R. (1998). DNA variation and the future of human genetics. Nat. Biotechnol..

[13] Weiss K.M., Terwilliger J.D. (2000). How many diseases does it take to map a gene with SNPs?. Nat. Genet..

[14] Liu N., Zhang K., Zhao H. (2008). Haplotype-association analysis. Adv. Genet..

[15] Almasi M., Zamani P., Mirhoseini S.Z., Moradi M.H. (2021). Genome-wide association study for postweaning weight traits in Lori-Bakhtiari sheep. Trop. Anim. Health Prod..

[16] Chen S., Zhou Y., Chen Y., Gu J. (2018). fastp: An ultra-fast all-in-one FASTQ preprocessor. Bioinformatics.

[17] Li H., Durbin R. (2009). Fast and accurate short read alignment with Burrows-Wheeler transform. Bioinformatics.

[18] Li H., Handsaker B., Wysoker A., Fennell T., Ruan J., Homer N., Marth G., Abecasis G., Durbin R. (2009). The Sequence Alignment/Map format and SAMtools. Bioinformatics.

[19] McKenna A., Hanna M., Banks E., Sivachenko A., Cibulskis K., Kernytsky A., Garimella K., Altshuler D., Gabriel S., Daly M. (2010). The Genome Analysis Toolkit: A MapReduce framework for analyzing next-generation DNA sequencing data. Genome Res..

[20] Wang K., Li M., Hakonarson H. (2010). ANNOVAR: Functional annotation of genetic variants from high-throughput sequencing data. Nucleic Acids Res..

[21] Purcell S., Neale B., Todd-Brown K., Thomas L., Ferreira M.A., Bender D., Maller J., Sklar P., de Bakker P.I., Daly M.J. (2007). PLINK: A tool set for whole-genome association and population-based linkage analyses. Am. J. Hum. Genet..

[22] Gondro C., Porto-Neto L.R., Lee S.H. (2013). R for genome-wide association studies. Methods Mol. Biol..

[23] Jiang L., Zheng Z., Qi T., Kemper K.E., Wray N.R., Visscher P.M., Yang J. (2019). A resource-efficient tool for mixed model association analysis of large-scale data. Nat. Genet..

[24] Wu T., Hu E., Xu S., Chen M., Guo P., Dai Z., Feng T., Zhou L., Tang W., Zhan L. (2021). clusterProfiler 4.0: A universal enrichment tool for interpreting omics data. Innovation.

[25] Barrett J.C. (2009). Haploview: Visualization and analysis of SNP genotype data. Cold Spring Harb. Protoc..

[26] Moser E.B., Saxton A.M., Geaghan J.P. (1988). Biological applications of the SAS system: An overview. Comput. Appl. Biosci. CABIOS.

[27] Craddock N., Sklar P. (2013). Genetics of bipolar disorder. Lancet.

[28] Jiang N., Wang Z., Guo X., Peng Z., He Y., Wang Q., Wu H., Cui Y. (2023). Hepatic Runx1t1 improves body fat index after endurance exercise in obese mice. Sci. Rep..

[29] Jiao Y., Huang B., Chen Y., Hong G., Xu J., Hu C., Wang C. (2018). Integrated Analyses Reveal Overexpressed Notch1 Promoting Porcine Satellite Cells’ Proliferation through Regulating the Cell Cycle. Int. J. Mol. Sci..

[30] Abe A., Yamamoto Y., Katsumi A., Okamoto A., Tokuda M., Inaguma Y., Yamamoto K., Yanada M., Kanie T., Tomita A. (2018). Rearrangement of VPS13B, a causative gene of Cohen syndrome, in a case of RUNX1-RUNX1T1 leukemia with t(8;12;21). Int. J. Hematol..

[31] Wu L., Gao L., Zhang D., Yao R., Huang Z., Du B., Wang Z., Xiao L., Li P., Li Y. (2018). C1QTNF1 attenuates angiotensin II-induced cardiac hypertrophy via activation of the AMPKa pathway. Free Radic. Biol. Med..

[32] Kim D., Park S.Y. (2019). C1q and TNF related protein 1 regulates expression of inflammatory genes in vascular smooth muscle cells. Genes Genom..

[33] Fukushima K., Nakamura A., Ueda H., Yuasa K., Yoshida K., Takeda S., Ikeda S. (2007). Activation and localization of matrix metalloproteinase-2 and -9 in the skeletal muscle of the muscular dystrophy dog (CXMDJ). BMC Musculoskelet. Disord..

[34] So K.K.H., Huang Y., Zhang S., Qiao Y., He L., Li Y., Chen X., Sham M.H., Sun H., Wang H. (2022). seRNA PAM controls skeletal muscle satellite cell proliferation and aging through trans regulation of Timp2 expression synergistically with Ddx5. Aging Cell.

[35] Yu M., Han S., Wang M., Han L., Huang Y., Bo P., Fang P., Zhang Z. (2022). Baicalin protects against insulin resistance and metabolic dysfunction through activation of GALR2/GLUT4 signaling. Phytomedicine.

[36] Leciejewska N., Pruszyńska-Oszmałek E., Mielnik K., Głowacki M., Lehmann T.P., Sassek M., Gawęda B., Szczepankiewicz D., Nowak K.W., Kołodziejski P.A. (2021). Spexin Promotes the Proliferation and Differentiation of C2C12 Cells In Vitro-The Effect of Exercise on SPX and SPX Receptor Expression in Skeletal Muscle In Vivo. Genes.

[37] Fang P., Zhang L., Yu M., Sheng Z., Shi M., Zhu Y., Zhang Z., Bo P. (2018). Activiated galanin receptor 2 attenuates insulin resistance in skeletal muscle of obese mice. Peptides.

[38] Lv X., Chen W., Wang S., Cao X., Yuan Z., Getachew T., Mwacharo J.M., Haile A., Sun W. (2023). Whole-genome resequencing of Dorper and Hu sheep to reveal selection signatures associated with important traits. Anim. Biotechnol..

[39] Zhang Q., Lee H.G., Han J.A., Kim E.B., Kang S.K., Yin J., Baik M., Shen Y., Kim S.H., Seo K.S. (2010). Differentially expressed proteins during fat accumulation in bovine skeletal muscle. Meat Sci..

[40] Liu R., Sun Y., Zhao G., Wang F., Wu D., Zheng M., Chen J., Zhang L., Hu Y., Wen J. (2013). Genome-wide association study identifies Loci and candidate genes for body composition and meat quality traits in Beijing-You chickens. PLoS ONE.

[41] Lu Y., Zhou J., Li F., Cao H., Zhang X., Yu D., He Z., Ji H., Lv K., Wu G. (2023). The Integration of Genome-Wide DNA Methylation and Transcriptomics Identifies the Potential Genes That Regulate the Development of Skeletal Muscles in Ducks. Int. J. Mol. Sci..

[42] Xu J., Shin J., McGee M., Unger S., Bando N., Sato J., Vandewouw M., Patel Y., Branson H.M., Paus T. (2022). Intake of mother’s milk by very-low-birth-weight infants and variation in DNA methylation of genes involved in neurodevelopment at 5.5 years of age. Am. J. Clin. Nutr..

[43] Hunsucker S.A., Spychala J., Mitchell B.S. (2001). Human cytosolic 5′-nucleotidase I: Characterization and role in nucleoside analog resistance. J. Biol. Chem..

[44] D’Ambrosio C., Hongo A., Li S., Baserga R. (1996). The role of Grb2 in the growth and transformation of mouse embryo cells. Oncogene.

[45] Maina F., Casagranda F., Audero E., Simeone A., Comoglio P.M., Klein R., Ponzetto C. (1996). Uncoupling of Grb2 from the Met receptor in vivo reveals complex roles in muscle development. Cell.

[46] Howey R., Cordell H.J. (2014). Imputation without doing imputation: A new method for the detection of non-genotyped causal variants. Genet. Epidemiol..

[47] Zhang L., Zhu Q., Liu Y., Gilbert E.R., Li D., Yin H., Wang Y., Yang Z., Wang Z., Yuan Y. (2015). Polymorphisms in the Perilipin Gene May Affect Carcass Traits of Chinese Meat-type Chickens. Asian-Australas. J. Anim. Sci..

